# Molecular Mechanisms Supporting Rice Germination and Coleoptile Elongation under Low Oxygen

**DOI:** 10.3390/plants9081037

**Published:** 2020-08-15

**Authors:** Chiara Pucciariello

**Affiliations:** PlantLab, Institute of Life Sciences, Scuola Superiore Sant’Anna, 56124 Pisa, Italy; chiara.pucciariello@santannapisa.it

**Keywords:** anoxia, coleoptile, flooding, hypoxia, rice, submergence

## Abstract

Rice germinates under submergence by exploiting the starch available in the endosperm and translocating sugars from source to sink organs. The availability of fermentable sugar under water allows germination with the protrusion of the coleoptile, which elongates rapidly and functions as a snorkel toward the air above. Depending on the variety, rice can produce a short or a long coleoptile. Longer length entails the involvement of a functional transport of auxin along the coleoptile. This paper is an overview of rice coleoptiles and the studies undertaken to understand its functioning and role under submergence.

## 1. Introduction

Submergence stress is one of the most critical constraints on crop production, with a significant impact on food security. Higher plants die rapidly when oxygen (O_2_) is limited due to water submergence. However, plants adapted to semi-aquatic environments are able to survive weeks of complete submergence by modifying their metabolism and morphology. Rice is known for its capacity to grow in paddy fields. However, in countries where rice is the predominant food, prolonged flooding can affect yield, since some varieties can be sensitive to complete submergence [1]. The rise of extreme weather events—with inundation and flash flooding—has caused considerable production losses.

In the last two decades, important adaptations have been identified with a significant impact on rice cultivation. The identification of the *SUBMERGENCE1* (*SUB1*) locus, responsible for limiting elongation during submergence in order to conserve energy, and its introgression into rice popular varieties [2,3], led to the development of SUB1 mega-varieties, which provide submergence tolerance with no effects on grain production and quality [4]. 

Rice seeds can germinate and elongate the coleoptile in the absence of O_2_. Other cereals such as wheat, barley, oat and rye lack this trait, and early experiments found it to be related to the capacity of rice to hydrolyse starch into readily fermentable sugars to generate ATP [5,6]. The energy produced by anaerobic fermentation is much lower to that produced in air. However, the reoxidation of NADH to NAD^+^ by ethanolic fermentation for use in glycolysis supports plant survival under O_2_ shortage.

The availability of α-amylase enzymes, which degrade starch under low O_2_, is among the key determinant of the germination of this species under submergence. ALPHA AMYLASE 3 (RAMY3D) is the only isoform significantly modulated under anoxia at the transcriptional level [7] and is regulated by sugar starvation [8], a condition occurring at the beginning of germination under O_2_-limiting conditions. This pathway enables rice grains to use the starchy reserves available in the endosperm in order to fuel the sink organs. 

During germination, rice elongates the coleoptile. The role of the coleoptile is to protect the true leaves during emergence from the soil and likely provide nutrients for the developing tissues [9]. Under submergence, the coleoptile acts like a snorkel that reaches the water surface and makes connection with the air [10]. The hollow structure allows a flow of O_2_ to travel down to underwater organs in order to establish aerobic respiration and fulfill the energy requirements of the developing leaves and roots. 

Together with coleoptile elongation, a delay in radicle emergence is observed under O_2_ shortage. This suggests that the development program is different under low O_2_ than in air. Under O_2_ shortage, the energy is preferentially allocated to fuel cell extension in the epigeal part of the plant and not to the root, which instead rapidly grow in air [10]. 

This article reviews how research into anaerobic rice coleoptiles has evolved and thus highlights the important role of this organ in rice seedling establishment under water. This trait may be of interest in areas particularly prone to flooding. Moreover, direct seeding associated with early flooding tolerance helps in suppressing weed growth and reducing the costs of mechanical and chemical weeding. 

## 2. The Coleoptile Elongation under Submergence

During aerobic germination, the rice coleoptile is initially white and turns green after one day [11]. The conical structure of the coleoptile is interrupted at the apex, where a vertical crack results in the following extension of true leaves [11]. The coleoptile generally has two vascular bundles running in parallel longitudinally. Some cell layers of the outer coleoptile epidermis develop chloroplasts during maturation, while the inner regions contain large amyloplasts [11]. Amyloplasts degrade rapidly in air with a complete decomposition at day 3, although the plastid membrane persists longer [12]. Along with the split in the coleoptile, aerenchyma is formed and senescence sets in [13]. In air, the energy supply from aerobic respiration, along with starch degradation in the endosperm, enables cell division and primary root development [14]. 

Under O_2_ shortage, there is no evidence of cell death events leading to coleoptile opening, aerenchyma formation and senescence [13]. Coleoptile maturation and cell death are blocked, and the coleoptile rapidly elongates while the root growth is dampened. The extension of the coleoptile under water occurs mainly through the cell expansion of cells preformed in the embryo [15], and the expansion increases with the time underwater [16]. On the contrary, the percentage of cells in mitotic phase decreases [16]. 

Indeed, both the length and the number of cells determine the final length of a coleoptile. However, cell division requires more energy than cell elongation, thus elongation is likely facilitated under O_2_ shortage [17]. Cell length increases from the base of the coleoptile with a peak one third of the way up [10]. The basal region of the hypoxic rice coleoptile seems to be the most intense for cell elongation and division.

When the submerged coleoptile reaches the water surface, the hollow structure of the coleoptile enables O_2_ to flow from the surface to the submerged parts. Coleoptile elongation is thus a strategy of the rice plant to avoid submergence stress and is referred to as the “snorkel effect” [18]. After reoxygenation, the rice plant shows a phenotype similar to that in air, since it will develop the primary root and leaf after coleoptile degeneration [14]. 

**Figure 1:**
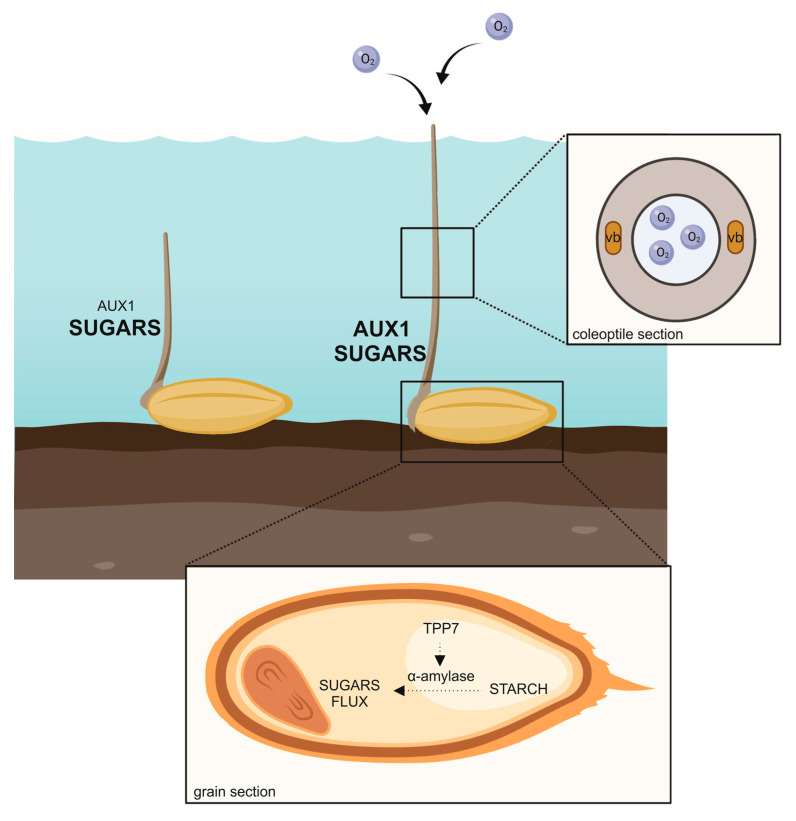
Rice elongates the coleoptile under water. Treahalose 6 phosphate phosphatase 7 (TPP7) availability in rice genotypes enables a better use of sugar, which is transferred from the source zone (endosperm) to the sink (embryo and coleoptile). Starch mobilization to fermentable sugars to fuel elongation takes place thanks to a battery of α-amylase whose transcription is activated by sugar starvation and oxygen shortage-dependent messages. Increased auxin transport by the influx carrier AUX1 contributes to a long coleoptile.

Under submergence, the rice coleoptile can grow several days without apparent senescence; it then reaches a plateau where cell extension is likely at a maximum [19,20]. At this plateau stage, different rice varieties show variability in coleoptile length, which can be a trait of genetic origin (Figure 1). Indeed, this condition can be considered specific for low O_2_ state, since aerobic coleoptiles degenerate after a few days. In this context, different water regimes in cultivation practices may have influenced the selection of genotypes with the capacity to reach a certain final coleoptile length as an adaptation.

## 3. Anaerobic Gene Regulation in Rice Coleoptile 

Analysis conducted on coleoptiles grown under anoxia until four days after germination, using the Nipponbare variety, revealed the regulation of the expression of several genes belonging to different families [7]. A detailed picture of this phenomenon in comparison to air was drawn, showing the upregulation of genes involved in the biochemical steps of pyruvate metabolism, glycolysis and fermentation. Interestingly, *RAMY3D* is strongly expressed in anoxic rice coleoptiles, besides the known role in the endosperm. The significance of this phenomenon is not known, but has been suggested to be a futile cycle of starch synthesis and degradation to fill amyloplasts with starch for the gravitropic response [7]. Another possibility is that starch is used as a provisional store derived from the degeneration of endosperm with the coleoptile representing a temporary sink of resources for the subsequent fast growth.

In parallel, the upregulation of *EXPA7* and *EXPA12* transcripts was observed. In this context, a subsequent study provided insights into the difference in expansin expression in rice varieties showing long (Arborio) and short (Lamone) coleoptile length [21]. No correlation was found between the transcriptional regulation of expansins and coleoptile elongation, suggesting that the differential elongation observed in the two varieties is not related to these genes. 

Genes coding for heat shock proteins (HSP) were also found to be induced in anoxic coleoptiles [7], in a mechanism that is devoted to the protection of plant cells under stress and that overlaps with the response to heat stress [22]. 

A general reduction in enzymes, whose activity requires O_2_, was observed [7], likely in an attempt to limit the energy spent on enzymes that are destined to be non-functional. In parallel, a possible sugar signalling role in genes induced under anoxia was observed, which the authors suggested was due to a moderate sugar starvation experience by anoxic rice coleoptiles. 

The study of cis-element enrichment in promoters of genes regulated in rice coleoptiles under anoxia related to glycolysis and fermentation [7] revealed the association with the transcription factors of ARF, ERF, MYB, WRKY and bZIP families [23]. In that study, the analysis of cis-element enrichment revealed the potential link with hormonal signalling, e.g., GA, IAA, ABA and ethylene. 

Dissimilarities in gene expression have been observed when comparing the basal with the tip coleoptile sections under anoxia and hypoxia [10]. These regions are characterised by a longitudinal steep gradient of growth, where the basal region is the most intense in terms of cell elongation and division. Some genes were found to be specifically expressed in the tip rather than the coleoptile base. A specific transcriptional program was also suggested to occur under anoxia and hypoxia. In anoxic coleoptiles, a specific expression of α-amylase genes has been identified at the base of the coleoptile in comparison to the tip [10]. 

Significant changes were observed in the epigenomic state of the coleoptile, with changes correlated to cell elongation during anaerobic conditions [14]. Very interestingly, the analysis in the DNA methylation observed in the reoxygenation phase after anaerobiosis showed a pattern similar to dry seeds rather than the pattern observed during exposure to air, suggesting that the plant’s internal clock is reset to react rapidly to molecular changes involved in root and true leaf elongation [14].

Hsu and Tung [24] conducted an RNA seq analysis of rice genotypes that have varying capacities to elongate the coleoptile under submergence. They assigned those genes that were significantly responsive in genotypes with rapid coleoptile growth, above all, to ethylene signalling and cell wall modification related pathways. 

## 4. Chromosomal Regions Regulating Coleoptile Elongation under Oxygen Shortage

Several works have recently been conducted to identify genomic regions associated with coleoptile elongation under water (Table 1). Screenings were performed considering rice panels composed of different varietal groups. Rapid coleoptile growth of submerged rice was studied in 153 rice accessions from *japonica* and *indica* varietal groups, measuring the difference in coleoptile length between control and submerged plants [25]. The authors used a Genome Wide Association Study (GWAS) and identified several genomic regions significantly associated with anaerobic germination. They also used a recombinant inbred line population obtained from a cross between *indica* and *japonica* varieties and identified a single significant Quantitative Trait Locus (QTL) region on chromosome 1 [25]. 

Zhang et al. investigated a pool of 432 *indica* varieties in terms of variations in the length of flooded coleoptiles, and used GWAS to detect significant single nucleotide polymorphisms (SNPs) [27]. One of the haplotypes of a candidate gene, coding for a DUF domain containing protein, contributed significantly to coleoptile elongation. 

A screening of over 8000 rice accessions performed at the International Rice Research Institute (IRRI) identified a few genotypes tolerant to anaerobic germination (AG) [26]. The QTL mapping of the backcross population obtained by the tolerant Khao Hlan On and the sensitive IR-64 identified five putative QTL on chromosome 1 (*qAG-3-1*), 3 (*qAG-3-1*), 7 (*qAG-7-2*) and 9 (*qAG-9-1* and *2*), which explained the phenotypic variation from 17.9 to 33.5%. The *OsTTP7* gene responsible for enhanced anaerobic germination was subsequently identified on *qAG-9-2* [29].

A further QTL mapping conducted using a population obtained from a cross between the sensitive cultivar IR42 and the tolerant Ma-Zhan Red identified six significant QTL on chromosome 2, 5, 6, and 7, with the largest effect on anaerobic germination played by *qAG-7.1* [2]. 

Our laboratory screened a panel of 273 *japonica* rice accessions for coleoptile length [20]. There were wide differences in coleoptile length, with a maximum length being reached at day 8 of dark submergence. This limited growth of the coleoptile can be due to energy restrictions by starch availability in the endosperm and release of soluble sugar or restrained capacity to sustain cell elongation over a certain limit. The interesting aspect of this panel was the high homogeneity [30], which led to the identification of new regions probably masked by high diversity in other panels. In this work, two marker trait associations (MTAs) were highly significant on chromosomes 1 and 5, with the identification of a subgroup of genes likely related to the coleoptile length trait. Of those genes, some related to auxin transport and sensing were found to be differentially expressed in long versus short coleoptile-harbouring varieties. In particular, the auxin transporter *AUX1* was found to be more expressed in rice varieties having a low ratio between the coleoptile length at day 8 on day 4, thus elongating very rapidly and showing a long coleoptile already at day 4. This gene was also more expressed in the group of rice varieties showing a long coleoptile at day 8.

Recently, chromosome segment substitution lines (CSSLs) obtained crossing Koshihikari variety, characterised by a long coleoptile, with IR 64 background, characterised by a short coleoptile, were evaluated for coleoptile elongation in anaerobic solution and tested in paddy field [28]. A novel QTL was identified on chromosome 3, referred to as *qACE3.1*, likely affecting the expression of genes involved in fermentative metabolism.

## 5. Starch Degradation during Anaerobic Rice Germination

The difference that makes rice stand out from other cereals is the use of the starch contained in the endosperm even under O_2_ shortage. Alpha amylase enzymes enable rice grains to degrade starch, which thereby allows the production of soluble sugars for sink organs. In air, these enzymes function through gibberellins (GA), abscisic acid (ABA) and sugar-demand mediated activation. In aerobic conditions, the α-amylases subfamily *AMY1* and *AMY2* are principally regulated by phytohormones GA and ABA [31]. The expression of α-amylase in air and anoxia differs in relation to where they are located. Under anoxia, the expression of total α-amylases transcripts decreases in aleurone and increases in embryo. In particular, the anoxia-dependent reduction of *AMY1* was observed with a simultaneous increase in *AMY3* [31].

Loreti et al. [8] investigated the *Tan-ginbozu* rice mutant, impaired in GA biosynthesis, and revealed that, under anoxia, the expression of α-amylase genes is independent of GA. Under O_2_ shortage, the subfamily *AMY3* is predominant [31], whose transcriptional regulation is mediated by sugar starvation and low O_2_ signalling [8]. Other cereals fail to degrade starch in low O_2_ conditions, due to the absence of these isoforms [6]. As a consequence, they cannot exploit the starchy reserves to activate the fermentation pathway and produce energy for growth.

Several studies have contributed to understanding the cascade effect that culminates with starch degradation by α-amylase. Rice plants mutated in a CALCINEURIN B-LIKE (CBL) INTERACTING PROTEIN KINASE 15 (CIPK15) *cipk15* were identified to be extremely sensitive to submergence during the early phase of development [32]. The *cipk15* mutant fails to elongate the coleoptile under low O_2_ and to express anaerobic genes (e.g., *ADH* and *AMY3*) [32]. In addition, a sucrose supply restored the *cipk15* phenotype under submergence, confirming the importance of sugar availability for this trait.

CIPK proteins are involved in decoding the Ca^2+^ signal sensed by CBL proteins, constituting a dual component for the Ca^2+^ sensing-responding system [33]. Although it is not clear how the mechanism functions under O_2_ deprivation, CBL4 was found to interact with CIPK15 [34] to positively regulate downstream events. On the contrary, CBL10 has been found to be a negative regulator of the CIPK15-dependent pathway since the maintenance of a low *CBL10* expression level in some rice varieties allows a higher expression of *AMY3* [35]. In the activation cascade, CIPK15 was shown to regulate the major sugar regulatory kinase SUCROSE NONFERMETNING 1-RELATED PROTEIN KINASE SnRK1A that subsequently activates the MYELOBLASTOSIS SUCROSE 1 (MYBS1) transcription factor involved in the transcriptional regulation of α-amylase genes [36,37,38]. Under sugar starvation, MYBS1 binds to the sugar response element located on the α-amylase promoter [37,38].

In the context of sugar availability, a panel of 141 Italian and 23 Sri Lankan rice varieties was tested for coleoptile length under anoxia [39]. Expansins were shown not to be involved in the coleoptile length difference among varieties [21], while the long coleoptile varieties showed a higher ethanol production, suggesting a better performance in fermentation in comparison to the short coleoptile varieties [39]. The *rice alcohol dehydrogenase 1-deficient* (*rad*) mutant showed a shorter coleoptile under low O_2_ in comparison to the wild-type [16]. This was mainly related to a reduced longitudinal cell length and repression of cell division in the coleoptile, together with ADH essentiality for fermentation in embryo and endosperm [40].

The TREHALOSE 6 PHOSPHATE PHOSPHATASE 7 (TPP7) gene, the genetic determinant of the major QTL *qAG-9-2* identified in the rice *japonica* variety Khao Hlan On [26], plays a key role in the mobilization of starch under water in terms of coleoptile elongation and embryo development [29]. This gene is absent in the variety IR64, which barely germinates and whose coleoptile does not elongate significantly under submergence. When *TPP7* gene is available, the dampening of the starch degradation due to a balance between trehalose 6 phosphate and sucrose likely decreases, and α-amylases can work toward the production of sugars for sink organs. The consumption of T6P with the production of trehalose by TPP7 removes the dampening effect of T6P on SnRK1A, activating the starch catabolism. TPP7 availability has been shown to contribute substantially to the elongation of coleoptiles. The isolation of the near isogenic line NIL-AG1 in the IR64 background showed an increase in coleoptile length and α-amylase activity [29]. The anaerobic germination sensitivity of IR64 was rescued by a sucrose supply, while it was not influenced by ABA or GA. The presence of haplotypes in a rice *japonica* panel characterised by the systematic presence of *TPP7* was shown not to influence coleoptile length [20].

## 6. Hormonal Regulation of Anaerobic Rice Coleoptile Elongation

The role of auxin in coleoptile development under submergence has been questioned for several years. There are contrasting data on the availability and role of auxin in anaerobic coleoptiles [41,42,43,44,45,46]. Early works reported that the treatment of IAA inhibitors in water resulted in dampened elongation growth [41,42]. During anaerobic treatment, an increase in the IAA level was found, which was suggested to be translocated from the endosperm [43]. However, the addition of IAA to coleoptiles under anoxia did not affect the final coleoptile length, unlike air where IAA enhances coleoptile elongation [44]. Subsequently, experiments in Heller medium flasks showed that IAA addition has an initial positive influence on the elongation of coleoptile segments, while the second step of elongation depends on ethylene [45]. The level of IAA was found to be lower in underwater rice segments than in air [46]. 

More recently, the submergence-dependent inhibition of *miR393* expression has been shown to reduce the degradation of the mRNA *TRANSPORT INHIBITOR RESPONSE 1* (*TIR1*), activating the auxin-dependent signalling pathway in hypoxic rice [47]. Under submergence, the auxin-dependent pathway induces coleoptile elongation and likely stomata development. *EXPA7* expression was found to be positively regulated by target-mimic lines of miR393, and negatively by miR393 overexpressing lines. Subsequently, *EXPA7* expression has been found to be significantly regulated in *japonica* rice varieties that have a long coleoptile [19]. A role for auxin availability and transport mediated by AUX1 has been identified in long coleoptile harbouring varieties [19]. In these varieties, the coleoptile tip likely induces a further production or redistribution of auxin along the coleoptile longitudinal axis that culminates in an increased length. 

There is a higher rate of ethylene production in rice genotypes that germinate and grow rapidly under low O_2_ [48]. Indeed, ethylene is a primary signal under submergence [49] and ethylene treatment increases the coleoptile length [44,50]. In rice, ethylene drives the expression of the SUB1A gene and the SNORKELs genes, which belong to the Ethylene Responsive Factor of group VII (ERF-VII) and help adult plants to tolerate submergence. In coleoptiles under anoxia, more ERFs are upregulated than in air, such as ERF60, ERF67, and ERF68 [7], which belong to the ERF-VII group, like SUB1 and SNORKEL genes. Group VII ERFs function as O_2_ sensors in Arabidopsis, and ERF66 and ERF67 have been identified as a target of SUB1A and, in contrast to SUB1A [51], of the N-end rule pathway [52]. In fact, a characteristic of ERF-VII is the conservation of the N-terminus, which promotes degradation in presence of O_2_ and stabilisation under hypoxia. This means that, under O_2_ shortage, ERF66 and ERF67 escape proteolysis and are stabilised for downstream transcriptional regulation of anaerobic genes [52]. 

Ethylene interacts with auxin in order to inhibit root elongation in rice seedling development [53] and it may extend the auxin-dependent elongation of rice coleoptiles under submergence [54]. It will be interesting to investigate how ERF-VIIs function in anaerobic coleoptiles and what gene targets they have in this organ. 

## 7. Conclusions

The ability to produce coleoptiles under hypoxia and anoxia, rather than in air, is a unique feature of rice. Other cereals fail to germinate under submergence. The involvement of sugar in this important trait has long been studied. Rice can hydrolase and use the starchy reserves available in the endosperm in times when O_2_ is absent. The cascade, which culminates with the activation of starch hydrolysing enzyme α-amylase, is not regulated by GA and ABA, like in air, but is modulated by sugar availability and low O_2_. The pathway has been explored in detail with the identification of key components, such as CIPK15, MYBS1 and SnRK1A. 

Rice varieties that have the *TPP7* gene are more effective than other varieties in moving sugars from source (endosperm) to sink (embryo and coleoptile). Of the phytohormones, the role of auxin in coleoptile length grown under water has long been controversial. Indeed, the discovery of miR393 regulation of *TIR1* transcription under submergence and the key role of auxin in long coleoptile open again the possibility that this hormone is involved in the trait. Under submergence, O_2_ is going down very rapidly [20]; however, a certain level is maintained in the first days and may support tryptophan-dependent auxin biosynthesis in germinating rice. Some authors hypothesised that the rice endosperm releases auxin during low O_2_. Rice grain contains IAA [19], which may be translocated to the elongating coleoptile. A key role in auxin transport has been proved by the phenotype of *osaux1* mutants, which shows a reduced coleoptile length under submergence in comparison to the background [19]. This result is very interesting also in relation to the possible auxin interaction with ethylene, as previously suggested, and the positive effect of ethylene on coleoptile growth. We know that the ethylene level increases under water and governs rice adaptation strategies to submergence. 

In this framework, many questions are still open: i) is the endosperm an auxin source in coleoptile elongation under submergence? ii) what is the auxin gradient in the submerged coleoptile? iii) is auxin involved in the delay of radicle emergence observed in submerged rice? iv) is there any interaction between auxin and ethylene in the regulation of coleoptile length under water? 

The answer to these questions should provide a comprehensive vision of the mechanisms involved in coleoptile elongation under water. This would help to find ways to develop coleoptile elongation in rice varieties that are needed for direct seeding or are cultivated in areas exposed to unexpected flooding. 

## Figures and Tables

**Table 1:** Chromosomal regions identified in genetic studies focused on rice germination and coleoptile elongation traits.

Trait of Study	Genotypes	Major Chromosomal Region	Reference
Tolerance to flooding during germination	Khao Hlan On backcross population with IR64 recurrent parent	Chr 9 *qAG-9-1* and *qAG-9-2* QTLs	Angaji et al., 2010 [26]
Tolerance to anaerobic conditions during germination	Population derived from a cross between Ma-Zhan Red and IR42	Chr 7 *qAG-7-1* QTL	Septiningsih et al., 2013 [2]
Coleoptile elongation under anaerobic germination	Recombinant inbred line population derived from a cross between *japonica* and *indica* varieties	Chr1 QTL	Hsu and Tung, 2015 [24]
Coleoptile length during germination under flooding	Panel of 432 *indica* rice varieties	Chr 6 MTAs	Zhang et al., 2017 [27]
Coleoptile length under dark submergence	Panel of 273 *japonica* rice accessions	Chr1, Chr5 MTAs	Nghi et al., 2019 [20]
Coleoptile length in anaerobic solution	39 chromosome segment substitution lines derived from a cross between Koshihikari and IR64 rice varieties	Chr3 *qACE3.1*	Noshimura et al., 2020 [28]
